# Cross-Cultural Adaptation and Validation of the Simplified Diabetes Knowledge Test (Arabic Version) for Insulin-Dependent Diabetic Patients: A Cross-Sectional Study in Iraq

**DOI:** 10.3390/jcm15031164

**Published:** 2026-02-02

**Authors:** Shaymaa Abdalwahed Abdulameer, Mohanad Naji Sahib

**Affiliations:** College of Pharmacy, Al-Farabi University, Baghdad 10022, Iraq

**Keywords:** cross-sectional, diabetic patient, Iraqi, simplified diabetes knowledge, translation, validation

## Abstract

**Background/Objectives**: Diabetes is major metabolic disorder and rapidly increasing public health problem globally. The greatest way to reduce diabetic complications is adequate knowledge about the condition. Hence, the primary objectives of this study were to evaluate the psychometric properties of the Simplified Diabetes Knowledge Test—Arabic version (SDKT-A) among Iraqi insulin-dependent diabetic patients. Additionally, the secondary objectives were to assess the associated independent variables and the risk of atherosclerosis and cardiovascular risk event by using atherogenic indices and lipid ratios with the SDKT-A. **Methods**: A cross-sectional, descriptive study was conducted in primary healthcare clinics. The SDKT was translated into Arabic using forward–backward translation, reconciliation, and pilot testing. Thereafter, psychometric properties of the SDKT-A were evaluated depending on different criteria. Atherogenic indices of Castelli risk indices I and II (CRI-I and II), triglyceride/HDL ratio, non-HDL-C ratio, atherogenic coefficient (AC), and triglyceride–total cholesterol–body weight index (TCBI) were calculated using specific formulas. **Results**: The SDKT-A questionnaire showed acceptable readability and validity. Cronbach’s alpha test (95% confidence interval) was 0.662 (0.59–0.73). The Pearson correlation coefficient of reliability for test–retest was found to be 0.659. The item difficulty index for most items was between 0.237 and 0.877. The point biserial correlation values ranged from 0.028 to 0.535 with Ferguson’s sigma value equal to 0.962. The content validation results showed a significant content validity ratio (CVR) value for most of the questions, ranging from 0.8 to 1. The content validity index (CVI) value for SDKT-A was found to be 0.98, which showed good agreement between experts. In addition, the exploratory factor analysis with promax rotation identified four domains for the final 20 items of the SDKT-A that explained 41.83% of the scale total variance. The mean score of the SDKT-A was 11.09 ± 3.40. The total score of the SDKT-A was positively and significantly correlated with education level (r = 0.322, *p* < 0.01). In addition, the total scores of the SDKT-A were negatively and significantly correlated with glycemic control, age, CRI-I, CRI-II, triglyceride/HDL ratio, AC, non-HDL-C ratio, and TCBI. Furthermore, the glycemic control (HbA1c) was positively and significantly correlated with the preventive measures factor (r = 0.175, *p* < 0.05), and were negatively and significantly correlated with the lifestyle and modification factor (r = −0.169, *p* < 0.05), diet and monitoring factor (r = −0.158, *p* < 0.05), and awareness factor (r = −0.149, *p* < 0.05). **Conclusions**: This study showed acceptable psychometric properties for the SDKT-A, with low levels of knowledge of diabetic disease in the sample population. Finally, comprehensive and interactive educational programs regarding lifestyle and modification, diet, and monitoring and awareness in primary healthcare centers in Iraq are warranted.

## 1. Introduction

Diabetes is major metabolic disorder and rapidly increasing public health problem globally that poses a significant serious health concern as it represents the fourth leading cause of mortality [1,2]. The International Diabetes Federation has anticipated that there will be an alarming rise in the prevalence of diabetes from 537 million (10.5%) in 2021 to 643 million (11.3%) and 783 million (12.2%) in 2030 and 2045, respectively [3,4]. This value considers diabetes as an epidemic and constituting significantly to morbidity with major chronic consequences [5]. Eastern Mediterranean and Middle-East (EMME) and North Africa have the highest regional prevalence rates with 9.2% in 2017, which will increase by 110% in 2045 [6]. In Iraq, the prevalence of diabetes ranged from 13.9% to 34% and it is estimated that around one and half million people have diabetes [7,8]. Many risk factors escalate the rise in the prevalence of diabetes and affect quality of life, including genetic and environmental predisposition, aging population, overweight or obesity, lack of physical activity, urbanization, economic status, and unhealthy diet [1,9]. These figures showed that the Arabic country is an endemic region with respect to diabetes. In Iraq, primary healthcare centers (PHCs) play an important role, as diabetic patients regularly (monthly base) visit these centers for essential medicines and supplies. All services offered in these PHCs are free with a waiver, with a list of medications and services offered at a discounted rate according to the Ministry of Health regulations. Despite the improvement in healthcare system services in the last two decades, this issue still represents a significant challenge affecting the capacity of the healthcare system and causes the awareness of people regarding their chronic diseases to become questionable. There are many factors that affect the quality of services, including a low number of PHCs, uneven physician and nurse distribution, health devices, medication availability, and limits to access and coverage [10,11].

The greatest way to reduce diabetic complications is adequate knowledge about the condition. It has been reported that awareness and knowledge of the disease can help people assess their risk, promote motivation to seek proper care, and encourage them to take charge of their self-management [12,13,14]. This could be accomplished by diabetic educators [15,16]. Most recent review articles regarding lifestyle medicine showed that the involvement of healthcare professionals in patient education regarding self-care will highly increase and improve health outcomes and management of the disease [17]. These programs or interventions were highly based on the starting level of patients’ knowledge and attitude to adapt lifestyle modification. It is well known that adequate diabetes knowledge has an impact on self-monitoring activities, which results in attenuating diabetic complications and reaching adequate glycemic control [18,19,20]. Alongside diabetes knowledge, other clinical factors also had an impact on self-care practice, like disease duration, type of treatment, and even the disease complications [21,22,23]. Therefore, studying patients’ knowledge and its associated factors will be vital for healthcare professionals in clinical decision-making and health support and attention [24]. Therefore, adapting a valid tool to assess patient knowledge is crucial.

A simplified Diabetes Knowledge Test (SDKT) is widely used and applicable for populations with limited literacy to reduce the difficulty of the previous DKT multiple-choice scale [25,26]. The SDKT was developed with (true/false/do not know) responses that make the SDKT easy and short to answer (20 items only) [27]. The Iraqi population’s literacy is variable between gender and location, which make this tool suitable for the low-education-level population [28]. Moreover, no study was conducted to evaluate the psychometric properties of the Arabic version of the SDKT among the Iraqi diabetic population. Thus, the research question is whether the SDKT is valid for Iraqi diabetic patients and what are the factors associated with diabetic knowledge among adults living with type 2 diabetes (T2D). Therefore, the primary objective of this study was to assess the psychometric properties of the SDKT (Arabic version, SDKT-A), while the secondary objectives were to evaluate its associated independent variables and atherogenic indices among Iraqi insulin-dependent T2D patients.

## 2. Materials and Methods

### 2.1. Research Design and Sampling Procedure

A cross-sectional, descriptive survey was conducted using the STROBE cross-sectional reporting guidelines (Supplementary File S1) [29] from November 2024 to March 2025 in primary healthcare clinics in Baghdad, Iraq. Through the random cluster sampling technique, six primary healthcare clinics from two large district zones (Al-Rusafa and Al-Kharkh) were selected. Equal numbers of patients (insulin-using patients) were recruited through a systemic random sampling method from each clinic after written informed consent. The inclusion criteria of diabetic patients were being older than eighteen years, having no mental problems, being able to write and read in Arabic, and registered as a diabetic patient in primary healthcare clinics for not less than one year. Each patient was asked to answer a questionnaire that included the socio-demographic data and the translated simplified Diabetes Knowledge Test—Arabic version (SDKT-A). The study protocol was approved by the Scientific Committee of the Al-Farabi University (02/2024-10).

To assess the validity and reliability of any tool, at least five to ten participants for each question are required for sample size calculation [30,31,32]. The SDKT English version contains 20 questions; hence, at least 200 patients were required for the validation process. However, for high precision and study power with the consideration of dropout, an estimated sample size was 300 patients. Although 300 patients agreed to participate in this study, 44 patients were excluded due to incompleteness or unavailability of laboratory data. Therefore, only 241 diabetic patients were eligible for this study. For the test–retest step, thirty-one patients were randomly recruited within 7 to 14 days according to the patients’ convenience.

### 2.2. Materials and Measurements

All patients completed the socio-demographic data and SDKT-A questionnaires. The original SDKT tool is 20 items with (true/false/do not know) responses (Supplementary File S2). The SDKT was obtained from the Michigan Diabetes Research Training Center [a project supported by the National Institute of Diabetes and Digestive and Kidney Diseases, Grant Number P30DK092926]. The possible total score ranges from 0 to 20 (the lowest value indicates a lower level of knowledge). A cut-off value of 13 was used to classify the SDKT-A total score value into low and high knowledge levels [27]. Anthropometric measurements were obtained for all patients, including height (cm), weight (kg), waist (cm), and hip (cm). Body mass index (BMI, the measure of obesity) was classified according to the World Health Organization [33].

Glycemic control was classified into good or optimal (<7), inadequate (7 to 8), and poor glycemic control (>8) according to the HbA1c measurement [34]. In addition, atherogenic indices and lipid ratios, including Castelli’s risk indices I (TC/HDL-c) and II (LDL-C/HDL-c) ratios, triglyceride/HDL ratio, non-HDL-c (TC minus HDL-c), atherogenic coefficient (AC), and the triglyceride–total cholesterol–body weight index (TCBI) were calculated using specific formulas to assess the risk of atherosclerosis and cardiovascular risk event. Low risk in atherogenic indices was defined as <3.5, <3.3 and, <3.0, <130 mg/dL, <3.0, and <985.3 for CRI-I, CRI-II, triglyceride/HDL ratio, non-HDL-c, AC, and TCBI, respectively [35,36,37].

### 2.3. Instrument Translation

According to international guidelines and previously published research, the SDKT was translated into Arabic using forward–backward translation, reconciliation, and pilot testing [38,39,40]. Two independent translators in Iraq undertook the translation process. Thereafter, five clinical pharmacists, three physicians, and the researchers served as an expert panel to evaluate the first SDKT-A version for reconciliation. Then, the reconciled version was back-translated to English by two different translators. Next, to settle any inconsistencies and harmonize the final version, repeated discussions between the translators and the expert panel were undertaken.

### 2.4. Validation

#### 2.4.1. Face and Content Validity

Ten experts in endocrinology and clinical pharmacy field were invited as subject matter experts to review, provide feedback, and judge the face validity of the translated questionnaire. [41]. Finally, fifteen patients from the same study demographic areas were recruited for a pilot study, and the tool was revised according to their comments. Quantitative content validity was assessed using the content validity ratio (CVR) and content validity index (CVI) [42]. The content validation was carried out by asking the expert panel whether or not the defined 20 items of the SDKT-A were “1 = essential”, “2 = useful but not essential” or “3 = not necessary”. According to Lawshe, with a panel of 10 experts, the minimum value of the CVR needs to be at least 0.62 to be acceptable and reach a significant level.

#### 2.4.2. Construct Validity

Exploratory factor analysis (EFA) was used to evaluate the construct validity of the SDKT-A. In EFA, the factor structure was established using the principal component factoring method for extraction with promax rotations for the SDKT-A. Factor analysis appropriateness of sampling adequacy was assessed by a Kaiser–Meyer–Olkin (KMO) value greater than 0.5, with a significance level less than 0.05 according to Bartlett’s test of sphericity. The number of factors retained was based on a combination of methods including Kaiser’s criterion (eigenvalue ≥ 1.0) and scree plot, as well as the conceptual meaningfulness of the rotated factors. A value of 0.40 was used as a viable cut-off point in judging the saliency of factor loadings [43].

#### 2.4.3. Reliability

Cronbach’s alpha (lowest acceptable value ≥ 0.50), corrected item total correlations (lowest acceptable value > 0.20) and Pearson’s correlation coefficient were used to measure reliability, internal consistency, and test–retest reliability, respectively [30,44].

#### 2.4.4. Item Difficulty Index

To measure the difficulty of answering the questions, the difficulty index (*p*) was assessed. The (*p*) is the proportion between the number of patients answering the item correctly to the total sample. The (*p*) value is a range between 0 and 1, and the most acceptable values are between 0.30 and 0.70 [45].

#### 2.4.5. Point Biserial Correlation and Discriminatory Power

The reliability index for each item was assessed by the point biserial correlation, which refers to an item’s total correlation. A correlation of <0.20 is considered poor [30]. In addition, to measure how broadly the total scores of a sample are distributed over the possible range, Ferguson’s sigma (discriminatory power) was measured. The item is considered discriminant if the value is above 0.9 [46].

### 2.5. Statistical Analysis

The results were presented as frequencies (percentages) and mean ± standard deviation (M ± SD). IBM SPSS Statistics software (version 26.0) with a significance level of < 0.05 was used. To assess the differences and correlations between variables, Kruskal–Wallis, Mann–Whitney U tests and Spearman rho were used, respectively. The psychometric properties comprised the assessment of item analysis, validation (face, content, and construct), and reliability (test–retest, and Cronbach’s alpha).

## 3. Results

### 3.1. Socio-Demographic and Diabetes-Related Information

Out of 210 patients, the mean age was 46.78 ± 15.92 years (range from 18 to 83 years). Females (115, 54.80%) outnumbered males (45.20%). The highest and lowest frequencies of patients were found in the age of less than 45 years (98, 46.70%) and more than 65 years (29, 13.80%), respectively. The average body mass index (BMI) was 27.93 ± 6.47 kg/m^2^, and most patients were overweight (81, 38.60%). The average waist-to-hip ratio was 0.87 ± 0.086. Most of the patients had a secondary school or lower level of education (124, 59.0%). The most common source of information about diabetes was from healthcare professionals (128, 61.00%). Other sources of information were social media and the internet (82, 39.0%). The average score of the SDKT-A was 11.09 ± 3.40 (which was considered low) and less than one-third of the sample population (59, 28.10%) had a high SDKT-A level. Furthermore, there was an insignificant difference in the SDKT-A scores between T1D (11.32 ± 3.21) and T2D (10.46 ± 3.83). Table 1 and Table 2 show the demographic data and diabetes-related variable results with the distribution of the two levels and the total diabetic knowledge.

More than half of the participants had a family history of diabetes (52.90%), a long duration of diabetes (more than ten years, 54.80%), and diabetic education in the last year (54.30%). Only 33.80% of diabetic patients had no comorbidities, and 35.30% of diabetic patients were hospitalized in the last year. Most of the patients had demonstrated poor (69.50%) and inadequate (16.70%) glycemic control. The mean ± standard deviation (median) and interquartile range (IQR) of HbA1c level among the T2D patients were 9.02 ± 2.15 (8.80), IQR: 7.40–10.20. The atherogenic indices (mean ± standard deviation (median)) were shown as follows: CRI-I (4.43 ± 1.94 (4)), CRI-II (2.68 ± 1.87 (2)), triglyceride/HDL ratio (4.44 ± 2.64 (4)), non-HDL (134.53 ± 50.87 (127.50)), AC (3.43 ± 1.94 (3)), and TCBI (2711.45 ± 2014.63 (2259)). The percentage of high CRI-I, CRI-II, triglyceride/HDL ratio, non-HDL-C ratio, AC, and TCBI in the sample population were 61.40%, 22.90%, 79.50%, 49%, 61,40%, and 91.40%, respectively. The atherogenic indices (CRI-I), triglyceride/HDL ratio, and AC were found to be significantly higher in the low-knowledge compared to high-knowledge diabetic patient groups (*p* < 0.05).

The total score of the SDKT-A was positively and significantly correlated with education level (r = 0.322, *p* < 0.01). In addition, the total score of the SDKT-A was negatively and significantly correlated with glycemic control (r = −0.160, *p* < 0.05), age (r = −0.233, *p* < 0.01), CRI-I (r = −0.213, *p* < 0.01), CRI-II (r = −0.219, *p* < 0.01), triglyceride/HDL ratio (r = −0.256, *p* < 0.01), AC (r = −0.213, *p* < 0.01), non-HDL-C ratio (r= −0.153, *p* < 0.05)), and TCBI (r = −0.197, *p* < 0.01). Furthermore, glycemic control (HbA1c) was positively and significantly correlated with the preventive measures factor (r = 0.175, *p* < 0.05) and was negatively and significantly correlated with the lifestyle and modification factor (r = −0.169, *p* < 0.05), diet and monitoring factor (r = −0.158, *p* < 0.05), and awareness factor (r = −0.149, *p* < 0.05).

The results showed significant differences between age groups, education levels, monthly income, smoking status, number of reflo-checks at home, and triglyceride level in relation to the total SDKT-A scores (all *p* < 0.05) (Table 1). In addition, there was a significant difference in the preference of patients regarding where to conduct the reflo-check and diabetic knowledge (*p* < 0.05) (Table 2).

The correct answer percentage of the SDKT-A is shown in Table 3. The results showed that the SDKT-A correct answer scores were variable. The total mean percentage of correct answers of the SDKT-A was 55.46 ± 18.74. The lowest percentage was 25.20% for “Lung problems are usually associated with having diabetes”, while the highest percentage of correct answers related to regular checkups and knowledge related to diabetes diet, with 82.40% and 81.10%, respectively. Moreover, less than 50% of correct answers were found within eleven items.

### 3.2. Validation

#### 3.2.1. Face Validation

The aim of the comprehensive translation processes and the pilot study was to make sure that the questionnaire words in Arabic language are the same in the English and/or that the alternative words have the same meaning and are easily understood by the Iraqi Arabic population. For example, item 2 contains the term “Glycosylated hemoglobin (HbA1c)”, which was not recognized by the translators or even the patients involved in the pilot study. Therefore, it was changed to “al-su-kar al-ta-ra-ku-me”, which means in English “cumulative sugar”. Moreover, regarding the item 3 question “A pound of chicken has more carbohydrate in it than a pound of potatoes”, the American population uses a pound for measuring weight while the Iraqi population is familiar with the kilogram, even though it is not equivalent in weight to a pound. Furthermore, less educated people will not recognize “carbohydrate (item 3)” or “unsweetened (item 6)” as the expert panel suggested. Therefore, they were changed to the words “Na-sha-we-yat” and “min gair sukar” (which are equivalent to starches and without added sugar in English, respectively). The word diet in item 7 was used as it is (“da-yat”), as the Iraqi population are familiar with this word’s meaning in its context. As a result, the SDKT-A questionnaire showed good readability as per feedback from the ten professionals, and the pilot study and the tool were ready for testing.

#### 3.2.2. Item Difficulty Index, Point Biserial Correlation, and Discriminatory Power

Table 3 represents the item analysis for the SDKT-A tool. Most items showed item difficulty index values within an acceptable range between 0.237 and 0.877. Only six items (1, 8, 9, 13, 18, and 19) scored above 0.75. Nevertheless, these items showed basic knowledge of diabetes, and the sample population was aware and knowledgeable of their context. Therefore, these six items were retained. No item was scored below 0.2 in the difficulty index level.

The point biserial correlation values ranged from 0.028 to 0.535. Although ten items showed values of less than 0.20, these questions seemed to be appropriate for retention as they measure personal diabetic knowledge. Moreover, the result showed an excellent Ferguson’s sigma value of 0.962.

#### 3.2.3. Content Validation

The Simplified Diabetes Knowledge Test—Arabic Version (SDKT-A) was evaluated by ten experts in the endocrinology, clinical pharmacy, and health education fields to identify and delete theoretically incoherent items, thus ensuring that the items in a scale demonstrate content adequacy. The results showed significant CVR values for the majority of the questions, ranging from 0.8 to 1, as shown in Table 4. A CVR value of 0.8 means those questions were considered essential by nine experts, whereas only one expert considered those questions “useful”, and no one considered any question “not necessary.” Similarly, a CVR value of 1 means those questions were considered essential by 10 experts, and no one considered any question “useful” or “not necessary.” The CVI value for SDKT-A was 0.98, which showed good agreement between experts and was calculated using the significant CVR of the retained items. Thus, it was inferred that all twenty variables were strongly valid for this research in its conceptual framework.

#### 3.2.4. Construct Validation

The exploratory factor analysis was conducted on the 20 items of the SDKT-A with promax rotation. The Kaiser–Meyer–Olkin measure verified the sampling adequacy for the analysis, KMO = 0.673, which indicated that the mediocre suitability of the data reduced a number of key factors, as it was greater than 0.5. The value of Bartlett’s test of sphericity was found to be highly significant (χ^2^ (190) = 718.401, *p* < 0.001). In addition, the EFA yielded four factors with eigenvalues greater than one, which explained 41.83% of the variance, as shown in Table 5.

**Figure 1:**
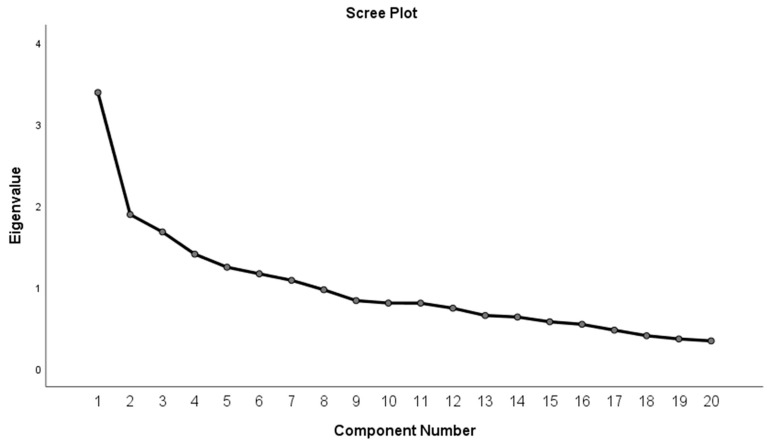
The scree plot of the Simplified Diabetes Knowledge Test—Arabic Version (SDKT-A).

The four factors in the SDKT-A were identified as factor 1 (lifestyle and modification), factor 2 (diet and monitoring), factor 3 (preventive measures), and factor 4 (awareness). The scree plot showed the point of inflection following the four factors, supporting the idea that four factor dimensions to measure knowledge may be appropriate for the SDKT-A, as shown in Figure 1. Regarding the four dimensions of the SDKT-A, there were positive correlations between factor 1 and both factors 2 and 4 (r = 0.267, *p* < 0.01; r = 0.165, *p* < 0.05, respectively). In addition, there was a negative correlation between glycemic control (HbA1c) and factors 1 (r = −0.169, *p* < 0.05), 2 (r = −0.158, *p* < 0.05), and 4 (r = −0.149, *p* < 0.05). However, there was a positive correlation between HbA1c and factor 3 (r = 0.175, *p* < 0.05). Reliability (Cronbach’s alpha) values for the four factors are shown in Table 5.

#### 3.2.5. Reliability

The scale had an overall Cronbach’s alpha (95% confidence interval) value of 0.662 (0.59–0.73), which is within the acceptable value range [44]. Moreover, the test–retest analysis showed that the Pearson correlation value was 0.659 (*p* < 0.01), and Cronbach’s alpha values for test–retest were 0.50 and 0.59, respectively. These results revealed that the tool had acceptable reliability and stable items for the Arabic version.

## 4. Discussion

Diabetes is one of the most global health problems with substantial morbidity and mortality. Approximately nearly a quarter million of adult individuals have undiagnosed diabetes, with almost half of these cases being unaware of their medical illness [4,47,48,49]. Moreover, diabetes has a significant financial impact on quality of life, wealth of individuals, and the healthcare system [50,51]. It is well known that good awareness and knowledge regarding diabetes could fill the gap in poor compliance, inadequacies in self-management, and glycemic control [52,53]. This could be accomplished by effective diabetic screening and educational programs, as early intervention could prevent diabetic complications. However, for all educational programs to be successfully implemented, patients’ needs and awareness must be assessed to empower diabetic patient self-monitoring and prevent diabetes-related complications [54]. Therefore, the cultural adaptation and evaluation of the psychometric properties of the SDKT (Arabic version) among Iraqi insulin-dependent diabetic patients was performed.

The validation and reliability results showed an acceptable face, content, and construct validity, internal reliability, and test–retest (stability over time) of the questionnaire. In the present study, the SDKT Arabic version scale comprises 20 items with four factors that explained 41.83% of the variance, which is consistent with other studies. Malaysian and United Arab Emirates studies showed that five factors explained 54.85% of the variance and three factors accounted for 41.2% of the variance [26,55]. The reason behind the differences in the number of dimensions between the present study and the Hasan S. et al. study [55] is the sample population. Hasan S. et al. used the tool among highly educated participants (70%), while our study demonstrated only about 41%. Hence, the present study results were more precise, as the tool developer suggested using it among low education levels. Moreover, four factors provided a clear picture of the gap in knowledge among low-education-level diabetic patients. Other studies assessed the Simplified Diabetes Knowledge (Arabic version) with either a low sample size and/or without a full assessment of psychometric properties [56,57]. Nonetheless, the present study fills the gap in psychometric assessment and cultural adaptation to ensure that the tool is culturally equivalent to the original Simplified DKT version.

The reliability of the SDKT-A was moderately consistent with an acceptable overall Cronbach’s alpha, which is comparable to the other studies [26,27,44,58]. The results showed that the participants may have some knowledge and awareness after 7 to 14 days of the re-test process, as Cronbach’s alpha for the reliability test was higher than the initial value. This result could be useful in measuring the changeover in the level of knowledge for the respondents within a range of time [54,59]. In addition, the difficulty indexes were within the acceptable level. Only six items (1, 8, 9, 13, 18, and 19) had values greater than 0.75. These items reflect good knowledge regarding diabetes diet, raised cholesterol, high blood pressure, foods low in fat, breakfast and blood glucose, and clinic appointments. This may be due to most of the respondents having a university education level. The most difficult items were 2 (glycosylated hemoglobin), 6 (unsweetened fruit juice), 10 (exercise), 12 (feet), and 15 (associated problems), as they are disease-specific questions. These items need to be addressed in any future diabetes education intervention. The same results were revealed with the original English version (four difficult items and four easy items to answer) [27]. These results depend entirely on the person’s underlying knowledge and item difficulty.

Moreover, point biserial correlation and Ferguson’s sigma values were within the acceptable range and indicated that the tool is reliable and discriminating. However, some items had low values in the point biserial correlation, but their deletion from the scale would not change Cronbach’s alpha significantly. Moreover, these items can fully describe a trait and the behavior of the patients; therefore, they appear to be good items to measure diabetic knowledge, and all of the items were related to the measured context in a coherent manner [30]. For better control and quality of life, the patients must be more knowledgeable about diabetes, complications, and its management [60,61]. Unfortunately, the present study showed a low overall knowledge score (11.09 ± 3.40). Comparable results regarding low diabetic knowledge were found in two Arabic population studies that used a revised version of the DKT [62,63].

The present study shows that more than two-thirds of diabetic patients (86.20%) had poor and inadequate diabetic control, in spite of nearly half of the patients (54.30%) receiving diabetic education regarding disease care and management and the noteworthy global advancements in diabetes education and treatment. Several studies revealed similar results regarding poor glycemic control [3,26,64]. Moreover, knowledge of diabetes varied with education level and smoking habit. As the patient becomes more educated and a non-smoker, knowledge increases. Conversely, another study showed that even with non-formal education for most patients, their knowledge of diabetes was high. This may be attributed to the active involvement of pharmacists and/or physicians in every scheduled healthcare appointment [65,66]. Nonetheless, a recent study regarding knowledge and practice toward chronic disease management among Iraqi pharmacists showed low levels in both aspects [67,68]. The current results were consistent with these studies, as the patients in the present study had little knowledge of T2D and low glycemic control (only 13.80% had good glycemic control). It is well known that diabetic educators and physician pharmacists collaboration toward the management of diabetes will improve glycemic control and patients’ knowledge about the disease [69,70]. The results showed that less than 40% of correct answers were in the dimensions of unsweetened fruit juice (item 6), exercise (item 10), foot care (item 12), associated problems (item 15), and clinic appointments (item 20). These results revealed questionable issues regarding patients’ healthy behaviors and attitudes toward diabetic management, which need to be addressed. Moreover, the reflo-check number, which represents one of the healthy behaviors toward diabetic management, can support these issues. The results showed that patients whose reflo-check was 5 or more a week before the study had higher knowledge. Moreover, patients’ preferences regarding where to conduct the reflo-check (private clinic and laboratory) enhances patients’ knowledge.

In the current study, correct responses regarding knowledge of risk factors, such as the used of glycosylated hemoglobin (HbA1c) to control blood glucose levels, was reported as 46.70%, and more than half of participants had poor glycemic control (69.50%). Moreover, this study found that patients with high education levels were significantly associated with a better knowledge score. This finding is consistent with other studies [71,72]. Alrashed FA et al. [73] showed that the patients with higher education levels had fewer severe comorbid diseases compared to those with lower education levels. As a result, the education level and finally the diabetic knowledge will have an impact on the disease prognoses and management. Moreover, the present study showed a negative association between lifestyle and modification, diet and monitoring, awareness, and the glycemic control. This supported by recent studies which highlight that lifestyle modification with exercise counseling program were vital element for comprehensive preventive management of T2D [74,75]. Additionally, the results showed that higher percentage of poor atherogenic indices among diabetic patients with low level of knowledge. Generally, dyslipidemia is known risk factors of coronary atherosclerosis and cardiac death [37,76]. Hence, the sample population may be at potential risk for developing atherosclerosis and cardiovascular disease (CVD). This conclusion was supported by other studies among Iraqi diabetic patients [77,78]. Furthermore, the sample population showed a good negative correlation between diabetes knowledge with HbA1c and atherogenic index data. All these variables are related to patients’ attitude which can be addressed in future study. An Iraqi study showed that enhancing diabetes knowledge was associated with increase healthy behavior of the diabetic patients through interactive educational program [79]. Other studies showed that the quality of life could be improved by enhancing diabetic knowledge through behavior change and metabolic control [52,80,81].

## 5. Study Limitation

The results cannot be generalized for all Arabic diabetic patients in Iraq as it was a cross-sectional and self-reported study. However, systemic and random cluster sampling methods with comprehensive translation, validity, and reliability steps revealed trusted results. Furthermore, future studies should be conducted in different types of hospitals with a larger sample size and/or multicenter study from different Arabic countries.

## 6. Conclusions

The current study revealed acceptable psychometric properties of the SDKT-A scale, which could be implemented in clinical settings. The correlation between tool dimensions, glycemic control, and atherogenic indices guides the healthcare professional to a personalized educational program and hence, their healthy behaviors. This study revealed a positive outlook of the cultural adaptation of the tool in the Iraqi population among low-education-level diabetic patients, as the developer suggested. Finally, comprehensive and interactive educational programs regarding lifestyle and modification, diet and monitoring, and awareness in primary healthcare centers in Iraq are warranted through addressing the underlining issues for low glycemic control and knowledge among insulin-dependent diabetic patients.

## Figures and Tables

**Table 1:** Demographic data with the distribution of the total diabetic knowledge and their two levels in the sample population, N = 210.

Variable	Total Sample Knowledge (N = 210) Mean ± SD (Median)	Low Knowledge (N = 151) Frequency (%)	High Knowledge (N = 59) Frequency (%)
**Age (years)**			
<45	11.96 ± 2.88 (12) *	67 (44.37)	31 (52.54)
45–54	10.15 ± 3.93 (10)	30 (19.86)	10 (16.94)
55–64	10.51 ± 3.44 (10)	32 (21.19)	11 (18.64)
≥65	10.31 ± 3.64 (10)	22 (14.56)	7 (11.86)
**Gender**			
Male	11.07 ± 3.26 (12)	71 (47.01)	24 (40.67)
Female	11.10 ± 3.53 (11)	80 (52.98)	35 (59.32)
**Marital Status**			
Married	10.86 ± 3.56 (11)	123 (81.45)	48 (81.35)
Single	12.10 ± 2.36 (12)	28 (18.54)	11 (18.64)
**Education level**			
No formal education	9.31 ± 3.31 (9.50) *	47 (31.12)	7 (11.86)
Secondary	11.16 ± 3.39 (12)	50 (33.11)	20 (33.89)
University	12.15 ± 3.02 (13)	54 (35.76)	32 (54.23)
**Employment status**			
Not employed	10.74 ± 3.43 (11)	88 (58.27)	33 (55.93)
Employed	11.57 ± 3.32 (12)	63 (41.72)	26 (44.06)
**Monthly income**			
No payment	12.02 ± 2.88 (13)	32 (21.19)	17 (28.81)
100,000–599,000 IQD	9.91 ± 3.55 (10) *	63 (41.72)	13 (22.03)
600,000–1,000,000 IQD	11.61 ± 3.28 (12)	56 (37.08)	29 (49.15)
**Smoking habit**			
Not smoking	11.45 ± 3.19 (12) *	123 (81.45)	53 (89.83)
Smoking	9.24 ± 3.90 (9)	28 (18.54)	6 (10.16)
**Body mass index (kg/m^2^)**			
Normal (18.5–22.9)	10.97 ± 3.31 (12)	51 (33.77)	15 (25.42)
Overweight (23–27.4)	11.11 ± 3.53 (11)	57 (37.74)	24 (40.67)
Obese (≥27.5)	11.19 ± 3.34 (12)	43 (28.47)	20 (33.89)
**Family History** **of diabetes**			
Yes	11.23 ± 3.46 (12)	77 (50.99)	34 (57.62)
No	10.94 ± 3.35 (11)	74 (49.00)	25 (42.37)
**Living area**			
Al-Kharkh	11.26 ± 3.33 (12)	82 (54.30)	35 (59.32)
Al-Rusafa	10.87 ± 3.50 (11)	69 (45.69)	24 (40.67)

* Significant level < 0.05; SD = standard deviation.

**Table 2:** Diabetes-related variables of the study population (N = 210).

Variable	Total Sample Knowledge (N = 210) Mean ± SD (Median)	Low Knowledge (N = 151) Frequency (%)	High Knowledge (N = 59) Frequency (%)
**Glycemic (HbA1c) control**			
Good control	12.28 ± 2.80 (13)	18 (11.9)	11 (18.60)
Inadequate control	12.51 ± 2.73 (13)	21 (13.90)	14 (23.72)
Poor control	10.51 ± 3.51 (11)	112 (74.20)	34 (57.60)
**Diabetes Duration (years)**			
<5	11.52 ± 3.60 (12)	16 (10.59)	9 (15.25)
5–9	10.69 ± 3.38 (11)	57 (37.74)	13 (22.03)
10–14	11.00 ± 3.29 (11)	39 (25.82)	16 (27.11)
15–19	10.61 ± 3.45 (11)	19 (12.58)	9 (15.25)
≥20	12.22 ± 3.36 (13)	20 (13.24)	12 (20.33)
**Hospitalization (last year)**			
Yes	10.69 ± 3.49 (11)	54 (35.76)	20 (33.89)
No	11.31 ± 3.34 (12)	97 (64.23)	39 (66.10)
**Type of insulin diabetes user**			
T1D	11.32 ± 3.21 (12)	109 (72.18)	45 (76.27)
T2D	10.46 ± 3.83 (11)	42 (27.81)	14 (23.72)
**Number of Co-morbidities**			
No Co-morbidities	11.66 ± 3.16 (12)	49 (32.45)	22 (37.28)
1 Co-morbidities	10.66 ± 3.83 (11)	43 (28.47)	13 (22.03)
2 Co-morbidities	10.76 ± 3.47 (10.5)	28 (18.54)	10 (16.94)
3 Co-morbidities	11.21 ± 2.81 (10.5)	10 (6.62)	4 (6.77)
4 or more Co-morbidities	10.90 ± 3.28 (11)	21 (13.91)	10 (16.94)
**Do you get diabetes education last year**			
Yes	11.51 ± 3.32 (12)	78 (51.65)	36 (61.01)
No	10.59 ± 3.45 (11)	73 (48.34)	23 (38.98)
**Visit physician at private clinic last year**			
Yes	11.18 ± 3.31 (12)	114 (75.49)	46 (77.96)
No	10.82 ± 3.70 (11)	37 (24.50)	13 (22.03)
**Number of reflo-checks at home (last week)**			
No	10.82 ± 2.81 (10)	17 (11.25)	5 (8.47)
once	10.32 ± 3.45 (10)	38 (25.16)	9 (15.25)
twice	11.17 ± 3.62 (12)	25 (16.55)	5 (8.47)
Three times	8.65 ± 3.12 (9)	16 (10.59)	1 (1.69)
Four times	10.20 ± 4.09 (11)	11 (7.28)	4 (6.77)
Five or more	12.29 ± 2.95 (13) *	44 (29.13)	35 (59.32)
**If you do not have glucose meter at home, you prefer to check blood glucose at:**			
Private clinic (reflo-check)	12.27 ± 3.62 (14) *^,^**	7 (4.63)	8 (13.55)
Pharmacy (reflo-check)	9.90 ± 3.51 (9)	39 (25.82)	10 (16.94)
Laboratory	11.89 ± 2.88 (12) *^,^**	74 (49.00)	34 (57.62)
Nurse (reflo-check)	9.89 ± 3.83 (11)	31 (20.52)	7 (11.86)
**TC**			
Good < 200 mg/dL	11.33 ± 3.28 (12)	109 (72.18)	44 (74.57)
Poor ≥ 200 mg/dL	10.46 ± 3.67 (11)	42 (27.81)	15 (25.42)
**HDL-C**			
Good ≥ 40 mg/dL	10.59 ± 3.51 (11)	63 (41.72)	18 (30.50)
Poor < 40 mg/dL	11.40 ± 3.30 (12)	88 (58.27)	41 (69.49)
**LDL-C**			
Good < 100 mg/dL	11.20 ± 3.22 (12)	86 (56.95)	33 (55.93)
Poor ≥ 130 mg/dL	10.95 ± 3.64 (12)	65 (43.04)	26 (44.06)
**TG**			
Good < 150 mg/dL	12.01 ± 2.84 (12) *	55 (36.42)	26 (44.06)
Poor ≥ 150 mg/dL	10.51 ± 3.60 (11)	96 (63.57)	33 (55.93)
**CRI-I**			
<3.5	11.90 ± 3.09 (13) *	51 (33.80)	30 (50.80)
≥3.5	10.58 ± 3.49 (11)	100 (66.20)	29 (49.20)
**CRI-II**			
<3.3	11.42 ± 3.16 (12)	115 (76.20)	47 (79.70)
≥3.3	9.98 ± 3.94 (10)	36 (23.80)	12 (20.30)
**Triglyceride/HDL ratio**			
3.0	12.47 ± 3.00 (13) *	23 (53.50)	20 (46.50)
≥3.0	10.74 ± 3.42 (11)	128 (76.60)	39 (23.40)
**non-HDL-c**			
<130 mg/dL	11.48 ± 3.24 (12)	72 (67.30)	35 (32.70)
≥130 mg/dL	10.69 ± 3.53 (11)	79 (76.70)	24 (23.30)
**AC**			
<3.0	11.90 ± 3.09 (13) *	51 (63.0)	30 (37.0)
≥3.0	10.58 ± 3.49 (11)	100 (77.50)	29 (22.50)
**TCBI**			
<985.3	13.11 ± 2.27 (13) *	10 (55.60)	8 (44.40)
≥985.3	10.90 ± 3.43 (11)	141 (73.40)	51 (26.60)

* Significant level between groups < 0.05, ** insignificant between groups; SD: standard deviation, %: percentage, HbA1c: glycated hemoglobin, T1D: type 1 diabetes, T2D: type 2 diabetes, TC: total cholesterol, HDL: high-density lipoprotein cholesterol, LDL: low-density lipoprotein cholesterol, TG: triglyceride, CRI-I: Castelli’s risk index I, CRI-II: Castelli’s risk index II, non-HDL: non-high density lipoprotein cholesterol, AC: atherogenic coefficient, TCBI: triglyceride–total cholesterol–body weight index of plasma.

**Table 3:** Psychometric properties of Simplified Diabetic Knowledge Test—Arabic version (SDKT-A) by Item Analysis.

Question Number	Correct Response (%)	Difficulty Index	Point Biserial Correlation †	Cronbach’s Alpha If Item Deleted
Question Number 1	81.10	0.763	0.132	0.661
Question Number 2	46.70	0.360	0.139	0.662
Question Number 3	71.90	0.719	0.204	0.654
Question Number 4	67.60	0.728	0.411	0.631
Question Number 5	46.70	0.465	0.109	0.666
Question Number 6	31.90	0.360	0.093	0.666
Question Number 7	49.50	0.544	0.306	0.642
Question Number 8	74.30	0.789	0.199	0.655
Question Number 9	78.10	0.851	0.115	0.663
Question Number 10	36.70	0.395	0.370	0.635
Question Number 11	47.10	0.474	0.446	0.625
Question Number 12	34.80	0.351	0.029	0.674
Question Number 13	77.60	0.877	0.406	0.633
Question Number 14	48.10	0.482	0.355	0.636
Question Number 15	25.20	0.237	0.028	0.672
Question Number 16	40.00	0.404	0.296	0.644
Question Number 17	57.60	0.544	0.470	0.622
Question Number 18	74.80	0.754	0.535	0.618
Question Number 19	82.40	0.851	0.127	0.661
Question Number 20	37.10	0.509	0.077	0.669

† Give corrected item total correlation.

**Table 4:** Content validity ratio (CVR) results for a simplified Diabetes Knowledge Test (SDKT)—Arabic version (N = 210).

No. of Question (Item)	ne	N	N/2	CVR	Interpretation
Question Number 1	10	10	5	1	Met the criteria for retention
Question Number 2	10	10	5	1	Met the criteria for retention
Question Number 3	10	10	5	1	Met the criteria for retention
Question Number 4	10	10	5	1	Met the criteria for retention
Question Number 5	10	10	5	1	Met the criteria for retention
Question Number 6	10	10	5	1	Met the criteria for retention
Question Number 7	10	10	5	1	Met the criteria for retention
Question Number 8	10	10	5	1	Met the criteria for retention
Question Number 9	10	10	5	1	Met the criteria for retention
Question Number 10	10	10	5	1	Met the criteria for retention
Question Number 11	10	10	5	1	Met the criteria for retention
Question Number 12	10	10	5	1	Met the criteria for retention
Question Number 13	10	10	5	1	Met the criteria for retention
Question Number 14	10	10	5	1	Met the criteria for retention
Question Number 15	9	10	5	0.8	Met the criteria for retention
Question Number 16	9	10	5	0.8	Met the criteria for retention
Question Number 17	10	10	5	1	Met the criteria for retention
Question Number 18	10	10	5	1	Met the criteria for retention
Question Number 19	10	10	5	1	Met the criteria for retention
Question Number 20	10	10	5	1	Met the criteria for retention

ne, the number of panelists indicating an item as essential; N, the total number of content experts in the panel (10); CVR, content validity ratio.

**Table 5:** Component matrix of exploratory factor analysis for the Simplified Diabetes Knowledge Test—Arabic Version (SDKT-A).

No. of Question (Item)	Factor 1	Factor 2	Factor 3	Factor 4	Communalities
Question Number 1		0.422			0.258
Question Number 2		0.600			0.347
Question Number 3		0.588			0.375
Question Number 4		0.520			0.452
Question Number 5		0.579			0.528
Question Number 6	0.295				0.265
Question Number 7	0.477				0.228
Question Number 8				0.605	0.504
Question Number 9				0.720	0.515
Question Number 10	0.614				0.382
Question Number 11	0.723				0.499
Question Number 12			0.735		0.577
Question Number 13	0.664				0.452
Question Number 14	0.547				0.342
Question Number 15		0.111			0.205
Question Number 16	0.599				0.405
Question Number 17		0.492			0.574
Question Number 18	0.602				0.524
Question Number 19			0.493		0.319
Question Number 20			0.786		0.614
Eigenvalues	3.390	1.892	1.678	1.406	
% of variance	16.950	9.459	8.390	7.029	Total = 41.83%
Reliability (Cronbach’s alpha)	α = 0.703	α = 0.504	α = 0.518	α = 0.485	Total scale: α= 0.662

## Data Availability

The original contributions presented in this study are included in the article. Further inquiries can be directed to the corresponding authors.

## Supplementary Materials

The following supporting information can be downloaded at: https://www.mdpi.com/article/10.3390/jcm15031164/s1, File S1: STROBE Statement—checklist of items that should be included in reports of observational studies; File S2: Simplified Diabetes Knowledge Test (SDKT).

## Abbreviations

The following abbreviations are used in this manuscript:

T2D	Type 2 Diabetes
T1D	Type 1 Diabetes
SDKT-A	Simplified Diabetes Knowledge Test—Arabic version
CVR	Content validity ratio
CVI	Content validity index
DKT	Diabetes Knowledge Test
