# Supplementary material for: Cross-Cultural Adaptation and Validation of the Simplified Diabetes Knowledge Test (Arabic Version) for Insulin-Dependent Diabetic Patients: A Cross-Sectional Study in Iraq
Source: J Clin Med. 2026 Feb 2;15(3):1164. doi: 10.3390/jcm15031164 (PMC12898498; doi:10.3390/jcm15031164)
Supplement: Supplementary file 1 [file jcm-15-01164-s001.zip › Supplementary file S2.pdf]

Research article: Cross-Cultural Adaptation and Validation of The Simplified Diabetes Knowledge Test (Arabic version) for Insu-lin-Dependent Diabetic Patients: A cross-sectional study in Iraq

Simplified Diabetes Knowledge Test (SDKT)

Question Number	Statements	Responses		
Question Number 1	The diabetes diet is a healthy diet for most people	True	False	Don't Know
Question Number 2	Glycosylated haemoglobin (HbA1c) is a test that measures your average blood glucose level in the past week	True	False	Don't Know
Question Number 3	A pound of chicken has more carbohydrate in it than a pound of potatoes	True	False	Don't Know
Question Number 4	Orange juice has more fat in it than low fat milk.	True	False	Don't Know
Question Number 5	Urine testing and blood testing are both equally as good for testing the level of blood glucose.	True	False	Don't Know
Question Number 6	Unsweetened fruit juice raises blood glucose levels.	True	False	Don't Know
Question Number 7	A can of diet soft drink can be used for treating low blood glucose levels	True	False	Don't Know
Question Number 8	Using olive oil in cooking can help prevent raised cholesterol in the blood.	True	False	Don't Know
Question Number 9	Exercising regularly can help reduce high blood pressure.	True	False	Don't Know
Question Number 10	For a person in good control exercising has no effect on blood sugar levels.	True	False	Don't Know
Question Number 11	Infection is likely to cause an increase in blood sugar levels.	True	False	Don't Know
Question Number 12	Wearing shoes a size bigger than usual helps prevent foot ulcers	True	False	Don't Know
Question Number 13	Eating foods lower in fat decreases your risk for heart disease.	True	False	Don't Know
Question Number 14	Numbness and tingling may be symptoms of nerve disease.	True	False	Don't Know
Question Number 15	Lung problems are usually associated with having diabetes	True	False	Don't Know
Question Number 16	When you are sick with the flu you should test for glucose more often.	True	False	Don't Know
**SKIP TO QUESTION 19 IF YOU DON'T TAKE INSULIN**				
Question Number 17	High blood glucose levels may be caused by too much insulin	True	False	Don't Know
Question Number 18	If you take your morning insulin but skip breakfast your blood glucose level will usually decrease.	True	False	Don't Know
Question Number 19	Having regular check-ups with your doctor can help spot the early signs of diabetes complications.	True	False	Don't Know
Question Number 20	Attending your diabetes appointments stops you getting diabetes complications	True	False	Don't Know
